# Combined Assessment of HPV and p16 Optimizes Clinical Decision‐Making in Head and Neck Cancer of Unknown Primary

**DOI:** 10.1002/cam4.72291

**Published:** 2026-09-14

**Authors:** Evelina Jörtsö, Anders Näsman, Igor Schliemann, Mark Zupancic, Lars Sivars, Tina Dalianis, Linda Marklund, Rusana Bark

**Affiliations:** ^1^ Department of Otorhinolaryngology Karolinska University Hospital Stockholm Sweden; ^2^ Department of Clinical Sciences Intervention and Technology, Division of ENT Diseases Karolinska Institutet Stockholm Sweden; ^3^ Department of Clinical Pathology Karolinska University Hospital Stockholm Sweden; ^4^ Department of Oncology‐Pathology Karolinska Institutet Stockholm Sweden; ^5^ Medical Unit Head, Neck, Lung, and Skin Cancer, Theme Cancer, Department of Head and Neck Surgery Karolinska University Hospital Stockholm Sweden; ^6^ Department of Immunology, Genetics and Pathology Uppsala University Uppsala Sweden; ^7^ Department of Surgical Sciences, Section of Otolaryngology and Head and Neck Surgery Uppsala University Uppsala Sweden

**Keywords:** cancer of unknown primary in head and neck, HNCUP, HPV, oropharyngeal cancer, p16

## Abstract

**Background:**

Human papillomavirus‐associated oropharyngeal squamous cell carcinoma (HPV+ OPSCC) is increasing in Sweden with implications for both the incidence and management of HPV+ head and neck cancer of unknown primary (HNCUP). In OPSCC, the combined presence of HPV DNA (HPV DNA+) and p16^INK4a^ (p16+) overexpression has been shown to provide superior prognostic information compared with either marker alone. In this study, we therefore aimed to assess whether the prognostic impact of HPV DNA and p16 concordance/discordance in HNCUP mirrors that observed in OPSCC, within a high HPV‐prevalent and low smoking population.

**Methods:**

In this retrospective population‐based cohort of 141 patients with HNCUP diagnosed at Karolinska University Hospital between 2000 and 2024, 128 had complete HPV DNA and p16 data and were included in analyses.

**Results:**

Consistent with previous findings in OPSCC, combined assessment of HPV DNA and p16 provided superior prognostic stratification compared with either marker alone. Nevertheless, HPV DNA+ as a single marker showed a prognostic impact almost comparable to that of combined HPV DNA+/p16+ status, while p16+ alone, although associated with some prognostic value, was less informative. HPV DNA‐negative disease was associated with poor prognosis irrespective of p16 status.

**Conclusions:**

The data suggest that combined assessment of HPV DNA and p16 is superior in HPV‐associated HNCUP. However, in settings where a single test must be chosen, and especially in areas with high prevalence of HPV + OPSCC, HPV DNA is preferable to p16. Such a strategy could simplify diagnostic workflows while still enabling effective risk stratification and supporting tailored patient management.

## 1 Introduction

Head and neck squamous cell carcinoma (HNSCC) often presents with metastatic cervical lymphadenopathy. When comprehensive evaluation fails to identify the primary tumor, the condition is classified as a head and neck cancer of unknown primary (HNCUP). The diagnostic work‐up of HNCUP is extensive and typically includes radiological imaging, fine‐needle aspiration cytology (FNAC), bilateral tonsillectomy, biopsies, and, at some centres, base‐of‐tongue mucosectomy [1, 2, 3, 4, 5, 6]. In this context, HNCUP accounts for 2%–5% of head and neck malignancies [7].

Of note, a considerable proportion of HNCUP cases are associated with human papillomavirus (HPV), with reported prevalence of HPV‐associated HNCUP varying between 22% and 63% across cohorts [8, 9, 10, 11]. Similar to oropharyngeal squamous cell carcinoma (OPSCC), HPV‐associated HNCUP has a more favorable prognosis, suggesting an HPV+ OPSCC origin in most cases [10, 12, 13, 14].

Overexpression of p16^INK4a^ (p16+) is currently used as a surrogate marker for HPV‐related disease and to determine TNM classification for OPSCC in the American Joint Committee on Cancer/Union for International Cancer Control (AJCC/UICC, 8th and 9th edition) staging system [15]. Similarly, HNCUP is classified in TNM8 and TNM9 according to whether the tumor is HPV‐related or not. However, in contrast to OPSCC, classification in HNCUP may rely on HPV DNA testing or p16 immunohistochemistry (IHC).

Previous studies have reported discordance between p16 and HPV DNA status in approximately 10%–26% of OPSCC cases. Moreover, patients with tumors positive for only one of these biomarkers had a less favorable prognosis compared to those with concordant HPV DNA+/p16+ tumors [16]. Consequently, reliance solely on p16 expression for TNM classification poses a risk of misclassification and potentially inappropriate treatment [17]. Furthermore, in accordance with findings in OPSCC, smaller studies suggest that discordant HPV DNA/p16 status may also occur in HNCUP, with potential implications for treatment stratification and survival outcomes [18].

In this study, we therefore aimed to assess whether the prevalence and prognostic impact of discordance between HPV DNA and p16 status in HNCUP mirror those observed in OPSCC in settings with high HPV‐prevalence and low smoking rate.

## 2 Patients, Material and Methods

### 2.1 Patient Characteristics and Selection

**FIGURE 1:**
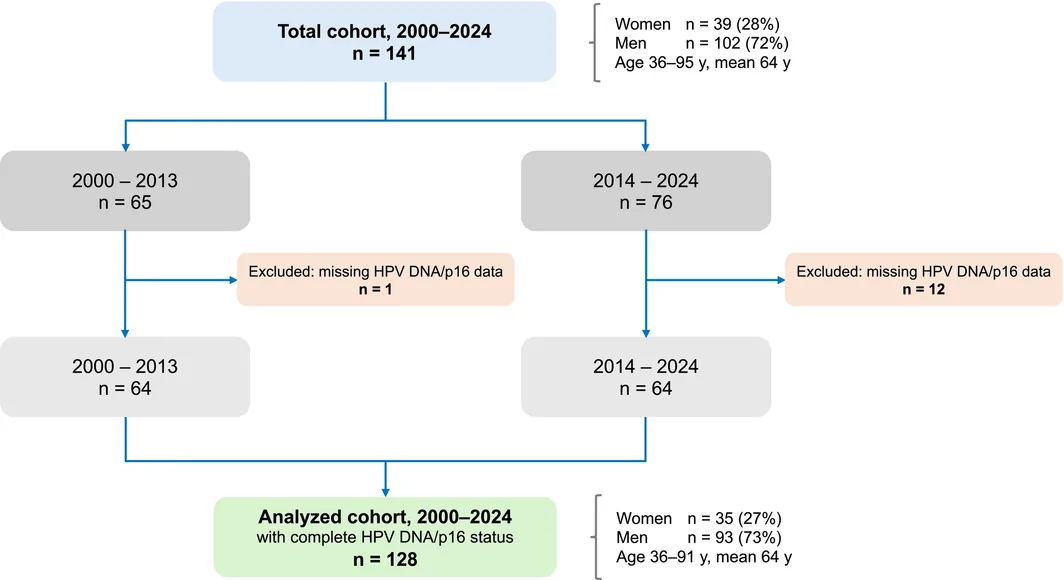
Flow diagram of patient selection and inclusion in the HNCUP cohort (2000–2024).

This retrospective, population‐based cohort study included all consecutively diagnosed patients with HNCUP at Karolinska University Hospital between 2000 and 2024 (*n* = 141). Of these, 128 patients had complete HPV DNA and p16 data and were included in the concordance analysis (Table 1 and Figure 1). All but three patients, all HPV DNA‐negative/p16 non‐overexpressing (HPV DNA−/p16‐) tumors, received curative‐intent treatment and were included in the final survival analyses (*n* = 125). Patients with missing p16 status (*n* = 13) did not differ from those included in the subsequent analyses with respect to TNM classification, sex, age, smoking status, treatment, or survival.

**TABLE 1:** Patient and tumor characteristics.

Characteristic	2000–2024 with complete HPV DNA/p16 status *n* = 128 (%)	2000–2013 *n* = 64 (%)	2014–2024 *n* = 64 (%)	*p* *
Sex				
Male	93 (72.7%)	48 (75.0%)	45 (70.3%)	0.692^a^
Female	35 (27.3%)	16 (25.0%)	19 (29.7%)	
Mean age, years (range)	64 (36–91)	63 (36–89)	66 (36–91)	0.124^d^
Smoking history				
Yes (Ever)^f^	90 (70.3%)	53 (82.8%)	37 (57.8%)	0.003^a^
No (Never)	38 (29.7%)	11 (17.2%)	27 (42.2%)	
*N* Stage (TNM7)				
N1	30 (23.4%)	16 (25.0%)	14 (21.9%)	0.367^e^
N2a	19 (14.8%)	12 (18.8%)	6 (9.4%)	
N2b	58 (45.3%)	26 (40.6%)	33 (51.6%)	
N2c	9 (7.0%)	3 (4.7%)	6 (9.4%)	
N3	12 (9.4%)	7 (10.9%)	5 (7.8%)	
*N* Stage				
III	30 (23.4%)	16 (25.0%)	14 (21.9%)	0.666^e^
IVA	85 (66.4%)	41 (64.1%)	44 (68.8%)	
IVB	12 (9.4%)	7 (10.9%)	5 (7.8%)	
IVC	1 (0.8%)	0 (0%)	1 (1.6%)	
HPV DNA				
Positive	71 (55.5%)	32 (50%)	39 (60.9)	0.286^b^
HPV16	62 (48.4%)	29 (45.3%)	33 (51.6%)	0.500^c^
HPV33	7 (5.5%)	2 (3.1%)	5 (7.8%)	
HPV33 + 58	1 (0.8%)	0 (0%)	1 (1.6%)	
HPV35	1 (0.8%)	1 (1.6%)	0 (0%)	
Negative	57 (44.5%)	32 (50.0%)	25 (39.1%)	
p16				
Positive	62 (48.4%)	33 (51.6%)	29 (45.3%)	0.596^a^
Negative	66 (51.6%)	31 (53.1%)	35 (54.7%)	
HPV DNA/p16				
HPV+/p16+	53 (41.4%)	29 (45.3%)	24 (37.5%)	0.026^e^
HPV+/p16—	18 (14.1%)	3 (4.7%)	15 (23.4%)	
HPV−/p16+	9 (7.0%)	4 (6.3%)	5 (7.8%)	
HPV−/p16—	48 (37.5%)	28 (43.8%)	20 (31.3%)	

Abbreviations: HPV, human papillomavirus; TNM, tumor, node metastasis.

^a^ Fisher's exact test, two‐tailed.

^b^ Fisher's exact test comparing HPV DNA+ vs. HPV DNA‐.

^c^ Fisher's exact test comparing HPV16 vs. other high risk HPV genotypes.

^d^ Independent *t*‐test, two‐sided.

^e^ Chi‐square test.

^f^ Ever smoker: current or former smoker (any history of cigarette smoking).

* Comparing the 2000–2013 and 2014–2024 cohorts.

Clinical data for patients diagnosed between 2000 and 2013, including HPV DNA and p16 status, were obtained from two previous studies by Sivars et al. [9, 19] Patients diagnosed between 2014 and 2024 were identified using NOMESCO surgical procedure codes UEN02, 05, 12, 15, UDH02, 05, UJC02, and ENB10.

Diagnosis and TNM staging were established by a multidisciplinary tumor board based on clinical evaluation, FNAC, computer tomography (CT), magnetic resonance imaging (MRI), and/or positron emission computer tomography‐computed tomography (PET‐CT). The seventh edition of the TNM classification system (TNM7) was applied to all patients. Patients diagnosed between 2017 and 2024 were retrospectively restaged according to TNM7 to ensure a uniform staging approach across the study cohort.

All patients underwent bilateral tonsillectomy and biopsies from the base of tongue and nasopharynx. Patient characteristics are summarized in Table 1.

### 2.2 Treatment Strategies Across the Study Period (2000–2013 vs. 2014–2024)

All patients diagnosed with HNCUP between 2000 and 2013, irrespective of tumor HPV status, as well as patients with HPV‐negative HNCUP between 2014 and 2024, were treated with elective neck dissection of the affected side followed by radiotherapy (RT) up to 68 Gy.

Between 2014 and 2024, patients with HPV‐associated HNCUP were treated with RT or chemoradiotherapy (CRT), in accordance with the treatment protocol for HPV‐associated OPSCC, followed by PET‐CT 3 months after completion of therapy. Neck dissection was performed in cases with suspected residual metastatic disease. After completion of treatment, patients were routinely followed with clinical examination every 3 months during the first 2 years and every 6 months thereafter for a total follow‐up period of 5 years.

### 2.3 Analysis of HPV DNA and p16 Expression

For patients diagnosed between 2000 and 2013, HPV DNA and p16 status were obtained from previous studies by Sivars et al. [9, 19] In this cohort, all analyses were performed on formalin‐fixed‐paraffin‐embedded (FFPE) samples obtained from the neck metastases. The presence of 27 HPV types was assessed using a bead‐based multiplex PCR assay (MagPix, Luminex Corp., Austin, TX, USA). For patients diagnosed between 2014 and 2024, HPV DNA analysis was performed on FNAC samples using the BD Onclarity HPV Assay (BD, Franklin Lakes, NJ, USA) according to clinical practice.

p16+ was defined as strong cytoplasmic and nuclear staining in > 70% of tumor cells. For patients diagnosed between 2000 and 2007, p16 expression was evaluated by IHC using the JC8 monoclonal antibody (Santa Cruz Biotech, Santa Cruz, CA, USA) on FFPE samples [9]. For all remaining cases, p16 status was assessed using the E6H4 clone (CINtec, Ventana, Tucson, AZ, USA). In patients diagnosed between 2014 and 2024, p16 status was assessed using FNAC samples.

In cases with missing data, complementary analyses were conducted when feasible, using frozen needle‐wash pellets (patients diagnosed between 2014 and 2024) or, when unavailable, on scraped cytological slides. p16 IHC was performed on cytological material using the same staining procedure as described above.

### 2.4 Statistical Analysis

Data pre‐processing, data presentation, and sample sizes are summarized in Table 1. All eligible patients were included in analyses. Continuous variables are presented as mean ± standard deviation (SD) or median (range), as appropriate, and categorical variables as number (percentage). No data transformation was applied, and no outliers were excluded.

Overall survival (OS) and disease‐free survival (DFS) were estimated using the Kaplan–Meier method (*n* = 138 for the HPV DNA analyses and *n* = 125 for the HPV DNA/p16 subgroup analyses). Three patients were excluded from the survival analyses because they received palliative‐intent treatment. Differences in survival between the groups were analyzed with the log‐rank test.

Univariable and multivariable Cox proportional hazards regression analyses were performed to estimate hazard ratios (*n* = 125). The proportional hazards assumption was assessed using Schoenfeld residual tests and graphical inspection of Schoenfeld residual plots. Global tests were performed for each model, and no significant violations of the proportional hazards assumption were detected.

Differences between groups were assessed using the chi‐square test, the independent *t*‐test, and Fisher's exact test (*n* = 128). All tests were two‐sided, and statistical significance was defined as *p* < 0.05. Statistical analyses were performed using IBM SPSS Statistics (Version 31.0; IBM Corp., Armonk, NY, USA) and R version 4.3.0 (R Core Team, Vienna, Austria).

## 3 Results

### 3.1 Presence of HPV DNA and p16 in HNCUP

All 141 HNCUP cases had sufficient material for HPV DNA analysis. Of these, 128 cases (64 patients diagnosed between 2000 and 2013 and 64 patients between 2014 and 2024) also had available material for p16 analysis and were therefore included in the subsequent analyses, while 13 cases were excluded due to incomplete biomarker data.

Within this cohort, 71/128 (55.5%) were HPV DNA+, of which 62/71 (87.3%) harbored HPV16, the predominant genotype. HPV33 was the second most common type. Although, the proportion of HPV DNA‐positive tumors increased from 32/64 (50.0%) in 2000–2013 to 39/64 (60.9%) in 2014–2024, and the proportion of HPV16 tended to decrease slightly from 29/32 (90.6%) to 33/39 (84.6%), none differences reached statistically significance (Table 1). In total, 62/128 (48.4%) of the cases were classified as p16+, and the proportions of p16+ tumors, i.e., 33/64 (51.6%) in 2000–2013 and 29/64 (45.3%) in 2014–2024, did not differ significantly (Table 1). Overall, 101/128 (78.9%) were HPV DNA/p16 concordant, including 53/128 (41.4%) HPV DNA+/p16+ and 48/128 (37.5%) HPV DNA−/p16‐ cases. Discordant profiles were observed in 27/128 patients (21.1%), comprising 9/128 (7.0%) HPV DNA−/p16+ and 18/128 (14.1%) HPV DNA+/p16‐ cases (Table 2).

**TABLE 2:** Prevalence of HPV DNA and p16 expression in patients with head and neck carcinoma of unknown primary (HNCUP).

	HPV DNA+	HPV DNA‐	Total	*p*
p16 overexpression	53	9	62	*p* < 0.0001
Non‐p16 overexpression	18	48	66	
Total	71	57	128	

*Note:* *p* < 0.0001 chi‐square test.

Abbreviation: HPV, human papillomavirus.

The median follow‐up time for the study cohort was 2514 days (approximately 6.9 years; range, 18–8557 days). Among the 125 patients treated with curative intent for whom both HPV DNA and p16 status were available, 39 deaths occurred during the follow‐up period. The number of deaths according to HPV DNA/p16 subgroup was as follows: HPV DNA+/p16+, 7 deaths (7/53, 13.2%); HPV DNA+/p16‐, 1 death (1/18, 5.6%); HPV DNA−/p16+, 5 deaths (5/9, 55.6%); and HPV DNA−/p16‐, 26 deaths (26/48, 54.2%).

Recurrence occurred in 35 patients. The number of recurrences according to HPV DNA/p16 subgroup was as follows: HPV DNA+/p16+, 6 recurrences (6/53, 11.3%); HPV DNA+/p16‐, 1 recurrence (1/18, 5.6%); HPV DNA−/p16+, 6 recurrences (6/9, 66.7%); and HPV DNA−/p16‐, 22 recurrences (22/48, 45.8%).

### 3.2 Prognostic Impact of Clinical Stage and Age, as well as HPV and p16 Status, Individually and in Combination, on Overall Survival (OS)

Advanced tumor stage was associated with worse OS. In the multivariable analysis, compared with Stage III disease, hazard ratios (HRs) were 3.78 (95% CI 1.14–12.56, *p* = 0.030) for stage IVA, 8.79 (95% CI 2.13–36.23, *p* = 0.003) for stage IVB, and 79.55 (95% CI 6.36–995.20, *p* < 0.001) for stage IVC. However, only one patient had stage IVC disease, limiting the interpretability for this estimate. Increasing age was also independently associated with poorer OS (HR 1.06 per year, 95% CI 1.02–1.10, *p* = 0.002) (Table 3a).

**TABLE 3:** Univariable and multivariable survival analyses in HNCUP.

Variable		HR	95% CI	*p*	HR	95% CI	*p*
a. Overall survival (OS)							
Stage	III	1 (ref)		< 0.001			0.001
	IVA	4.093	1.25–13.44	0.20	3.78	1.14–12.56	0.03
	IVB	9.39	2.34–37.66	0.002	8.79	2.13–36.23	0.003
Age		1.06	1.03–1.09	< 0.001	1.06	1.02–1.10	0.002
Smoking	Ever^a^	2.68	1.12–6.40	0.026	2.29	0.95–5.56	0.066
	Never	1 (Ref)					
HPV DNA/p16 status	HPV+/p16+	1 (Ref)		< 0.001			0.002
	HPV+/ p16—	0.43	0.05–3.48	0.427	0.31	0.04–2.55	0.274
	HPV−/p16+	5.62	1.78–17.73	0.003	4.76	1.48–15.36	0.009
	HPV−/p16—	5.95	2.58–13.75	< 0.001	3.65	1.51–8.85	0.004
b. Disease‐free survival (DFS)							
Stage	III	1 (ref)		0.005			0.003
	IVA	4.34	1.01–18.65	0.048	4.02	0.93–17.35	0.062
	IVB	15.24	3.06–75.96	< 0.001	18.26	3.50–95.19	< 0.001
Age		1.03	0.99–1.06	0.15	0.99	0.95–1.04	0.719
Smoking	Ever	2.91	1.01–8.43	0.05	3.13	1.04–9.38	0.042
	Never	1 (Ref)					
HPV DNA/p16status	HPV+/p16+	1 (Ref)		0.007			0.012
	HPV+/p16—	3.02	0.76–12.08	0.118	4.09	0.96–17.43	0.057
	HPV−/p16+	6.00	1.34–26.84	0.019	5.17	1.14–23.57	0.033
	HPV−/p16—	6.55	2.18–19.68	< 0.001	6.78	2.17–21.21	0.001

Abbreviations: HNCUP, head and neck carcinoma of unknown primary; HPV, human papillomavirus; HR, hazard ratio; Ref, reference.

^a^ Ever smoker: current or former smoker (any history of cigarette smoking).

**FIGURE 2:**
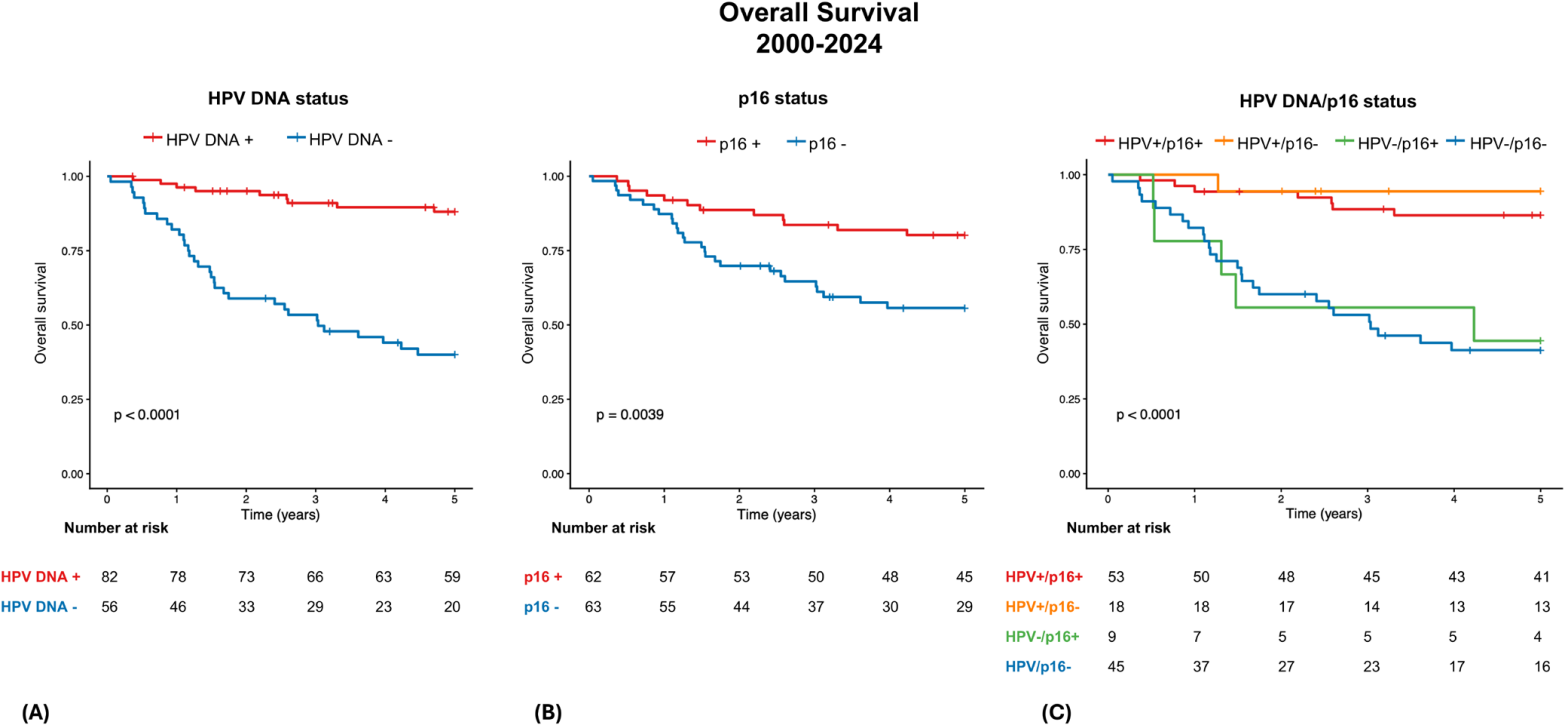
Overall survival for HNCUP divided by HPV DNA/p16 status, visualized with Kaplan–Meier curves. (A) Kaplan–Meier curves according to only HPV DNA status. HPV DNA‐ is marked blue, HPV DNA+ is marked red. (B) Kaplan–Meier curves according to only p16 status. p16‐ is marked blue, p16+ is marked red. (C) Kaplan–Meier curves when combining the two biomarkers, HPV DNA and p16, yielding four subgroups. HPV+/p16+ is marked red, HPV+/p16‐ is marked orange, HPV−/p16+ is marked green, HPV−/p16‐ is markd blue.

HPV DNA/p16 status was strongly associated with OS. Patients with HPV DNA+/p16+ tumors, irrespective of stage, age and smoking, demonstrated the most favorable outcomes and were used as the reference group. Both groups with HPV DNA‐negative HNCUP (HPV DNA−/p16+ and HPV DNA−/p16‐) had significantly worse OS in the multivariable analysis, with HRs of 4.76 (95% CI 1.48–15.36, *p* = 0.009) and 3.65 (95% CI 1.51–8.85, *p* = 0.004), respectively. This indicates that HPV DNA‐ HNCUP is associated with poorer survival, even in p16+ cases. In contrast, OS in patients with HPV DNA+/p16‐ tumors did not differ significantly from the reference group (HR 0.31, 95% CI 0.04–2.55, *p* = 0.274). Notably, Kaplan–Meier analysis suggested even higher OS in this group compared to HPV DNA+/p16+ tumors (Table 3a and Figure 2). Although some visual crossing of Kaplan–Meier curves was observed, the proportional hazards assumption was not violated according to Schoenfeld residual testing (OS global *p* = 0.94).

### 3.3 Prognostic Impact of Clinical Stage and Age, as well as HPV and p16 Status, Individually and in Combination, on Disease‐Free Survival (DFS)

Advanced stage was associated with a higher risk of recurrence. In multivariable analysis, using Stage III as a reference group, HRs were 4.02 (95% CI 0.93–17.35, *p* = 0.062) for stage IVA and 18.26 (95% CI 3.50–95.19, *p* < 0.001) for stage IVB (Table 3b). As stated above, stage IVC comprised only one patient; consequently, these results could not be interpreted further. Age was not significantly associated with DFS in either univariable HR 1.03 (95% CI 0.99–1.06, *p* = 0.15) and multivariable (HR 0.99, 95% CI 0.95–1.04, *p* = 0.719) analysis. The proportional hazards assumption was formally assessed using Schoenfeld residual tests. No evidence of violation was detected for the DFS model (global test *p* = 0.098).

**FIGURE 3:**
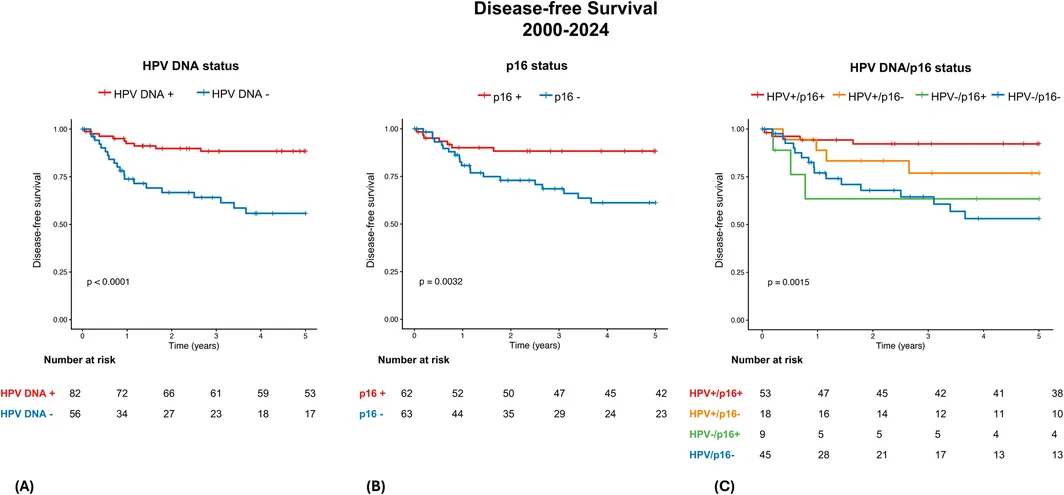
Disease‐free survival for HNCUP divided by HPV DNA/p16 status, visualized with Kaplan–Meier curves. (A) Kaplan–Meier curves according to only HPV DNA status. HPV DNA‐ is marked blue, HPV DNA+ is marked red. (B) Kaplan–Meier curves according to only p16 status. p16‐ is marked blue, p16+ is marked red. (C) Kaplan–Meier curves when combining the two biomarkers, HPV DNA and p16. HPV+/p16+ is marked red, HPV+/p16‐ is marked orange, HPV−/p16+ is marked green, HPV−/p16‐ is markd blue.

HPV DNA/p16 status predicted DFS similarly to OS. Patients with HPV DNA+/p16+ tumors had the lowest risk of recurrence and were set as the reference group. Patients with HPV DNA−/p16+ and HPV DNA−/p16‐ tumors had similarly increased risk of recurrence, with HRs of 5.17 (95% CI 1.14–23.57, *p* = 0.033) and 6.78 (95% CI 2.17–21.21, *p* = 0.001), respectively. Patients with HPV DNA+/p16‐ tumors showed a non‐significant increase in recurrence risk, although not reaching statistical significance (HR 4.09, 95% CI 0.96–17.43, *p* = 0.057) (Table 3b and Figure 3).

### 3.4 Survival Outcomes in Relation to Smoking Status

Over the study period, the proportion of ever‐smokers within the cohort decreased significantly from 53/64 (82.8%) in 2000–2013 to 37/64 (57.8%) in 2014–2024, *p* = 0.003 (Table 1). Nevertheless, smoking status was associated with survival outcomes. In univariable Cox regression, ever‐smokers had a higher risk of death compared with never‐smokers (HR 2.68, 95% CI 1.12–6.40, *p* = 0.026) and an increased risk of recurrence (HR 2.91, 95% CI 1.01–8.43, *p* = 0.050).

In multivariable models adjusting for age, stage, and HPV DNA/p16 status, smoking was no longer significantly associated with OS (HR 2.29, 95% CI 0.95–5.56, *p* = 0.066) but remained an independent predictor of worse DFS (HR 3.13, 95% CI 1.04–9.38, *p* = 0.042) (Table 3a,b).

### 3.5 Survival Outcomes in Relation to HPV‐Genotype

Both OS (*p* < 0.001) and DFS (*p* = 0.006) differed significantly according to HPV genotype, with both HPV16‐positive and non‐HPV16‐positive tumors demonstrating more favorable outcomes than HPV‐negative tumors. However, the number of non‐HPV16 cases was small, precluding more detailed genotype‐specific analyses or meaningful comparisons between individual HPV‐genotypes.

## 4 Discussion

This present study of HNCUP patients in a region with high HPV prevalence and low smoking rates provides further evidence supporting the prognostic value of combined HPV DNA and p16 status. We found that HPV DNA+ HNCUP, particularly when combined with p16+, was consistently associated with favorable survival outcomes, whereas HPV DNA‐ HNCUP, regardless of p16 status, exhibited a markedly poorer prognosis.

These findings were to some extent in contrast with those from lower HPV‐prevalence cohorts, where other experimental approaches were utilized and where p16+ status was suggested to be a favorable factor [12, 13, 20]. In this high HPB‐prevalence cohort, our findings instead indicate that p16+ without concomitant HPV DNA detection may be insufficient for reliable prognostication in HNCUP, and reinforce the prognostic importance of HPV DNA testing alongside p16.

Of note, the annual number of HNCUP cases remained relatively stable throughout the study period, in contrast to the increasing incidence of OPSCC, suggesting improvements in the diagnostic work‐up over time. Since 2014, HPV DNA analysis has been routinely performed on neck metastases in the Stockholm region, and HPV DNA+ HNCUP have been clinically managed similarly to HPV‐associated OPSCC [21]. Moreover, to improve detection of the primary tumor, some centres perform base‐of‐tongue mucosectomy, often using trans oral robotic surgery (TORS) or transoral laser microsurgery (TLM) [3, 22, 23]. While this approach may increase the identification of occult primary tumors, it is associated with an increased risk of increased morbidity, particularly postoperative dysphagia, without any corresponding improvement in survival [24].

In this study, the prevalences of HPV DNA+, p16+ and HPV DNA+/p16+ were 55.5%, 48.4% and 41.4%, respectively. These data are consistent with previous reports from other HNCUP cohorts from geographical areas with a high HPV DNA+/p16+ prevalence [7, 11, 12]. In contrast, other European cohorts with lower HPV‐prevalence, displayed markedly lower HPV DNA+/p16+ prevalence, ranging from 18% to 26%, along with lower proportions of HPV DNA+ and p16+ when assessed individually (approximately 20% and 30%, respectively) [13, 20]. Furthermore, the degree of HPV DNA/p16 discordance varied between HNCUP cohorts and appeared to be lower in cohorts with high HPV DNA+ and p16+ prevalences, with a reported range of 15%–48% across high‐ versus low‐prevalence settings [11, 13, 20]. Here, discordance was observed in 21.1% of cases, consistent with findings in other cohorts with high prevalence of HPV DNA and p16 positivity. Collectively, these findings indicate that HPV DNA/p16 discordance is a reproducible feature of HNCUP, albeit with considerable inter‐cohort variability, and underscores the importance of dual‐marker strategies to ensure accurate biological classification and to guide clinical treatment.

Previous studies in HNCUP have demonstrated improved survival associated with HPV DNA+ and p16+ when these biomarkers were analyzed separately, suggesting their role as independent prognostic indicators [10, 11, 12, 14, 19, 20]. Here, HPV DNA+ emerged as a strong predictor of improved OS and DFS. However, the combined HPV DNA/p16 classification further elucidated distinct prognostic subgroups; HPV DNA+/p16‐ cases exhibited OS outcomes comparable to HPV DNA+/p16+ cases, whereas HPV DNA‐ subgroups showed markedly poorer prognosis regardless of p16 status. Similarly, patients with HPV DNA+/p16‐ tumors demonstrated better DFS than the HPV DNA‐ subgroups; however, the point estimate relative to the HPV DNA+/p16+ group indicated a higher recurrence risk (HR 4.09, 95% CI 0.96–17.43), an estimate based on a single recurrence event and therefore highly imprecise.

In this study, p16+ alone did not reliably identify patients with a favorable survival. The observed divergence in the prognostic impact of HPV DNA and p16 expression across HNCUP cohorts underscores the need for cohort‐specific evaluation of these markers. Consequently, before defining clinical testing strategies, it is essential to determine which marker, or combination of markers, most accurately identifies patients with a favorable prognosis within the local population. Assessment of both HPV DNA and p16 is preferable when feasible. However, in some settings where only one marker can be assessed, particularly in high HPV‐prevalence cohorts, HPV DNA testing alone may provide more reliable prognostic stratification than p16 assessment alone.

Variation in smoking exposure across HNCUP cohorts likely contributes to heterogeneity in the reported survival associations for HPV DNA, p16, and their combinations. Populations with a higher tobacco burden generally show lower HPV prevalence, greater HPV DNA/p16 discordance, and a weaker or less consistent survival benefit associated with p16 + [13]. In contrast, cohorts with lower smoking exposure more consistently demonstrate improved outcomes even when HPV DNA and p16 are analyzed separately [11, 12]. These differences highlight the clinical value of dual‐marker assessment to improve prognostic precision.

In this study, the proportion of ever‐smokers tended to decrease over time, coinciding with a numerical increase in the prevalence of HPV‐associated tumors. Smoking emerged as a potential factor affecting biomarker discordance and prognosis. Low smoking‐exposure cohorts report moderate discordance rates (approximately 15%–20%) [11, 12], whereas cohorts with heavily smoking exposure show substantially higher rates, often with p16+ tumors lacking detectable HPV [13, 20]. This suggests that tobacco‐related carcinogenesis may induce p16 overexpression independently of HPV, thereby reducing its prognostic specificity as a surrogate marker and complicating tumor classification in HNCUP.

In our cohort, patients with HPV DNA+/p16+ tumors and low tobacco exposure consistently demonstrated the most favorable outcomes. This aligns with studies reporting that heavy smoking attenuates the survival advantage associated with HPV DNA or p16 positivity in HNCUP [11, 12, 13, 20]. Together, these findings suggest that smoking status should be considered in survival analyses alongside HPV DNA and p16 status, provided that smoking exposure is accurately documented.

Of note, the proportion of HPV DNA+ HNCUP was lower than that reported for OPSCC in the same region during the same time period and was associated with a higher proportion of discordant cases [16, 18]. More specifically, in the present HNCUP cohort, the prevalences of HPV DNA and p16 exceeded those observed in non‐tonsillar OPSCC but were lower than those observed in tonsillar and base‐of‐tongue cancer. Previous studies have demonstrated high concordance between HPV DNA and p16 in tonsillar and base‐of‐tongue cancer, with marker discordance as low as approximately 10%, whereas discordance approached 50% in non‐tonsillar/base‐of‐tongue OPSCC, reducing the reliability of p16 as a surrogate marker for HPV in these tumors [16, 17]. These findings are also consistent with our unpublished data from the corresponding Stockholm cohort (2000–2022), despite differences in the study periods. Within this spectrum, HNCUP appears to occupy an intermediate entity [18]. In line with previous reports, a relatively high degree of HPV DNA/p16 discordance 27/128 (21.1%) was observed in our cohort, further underscoring the limitations of single‐marker classification.

Nevertheless, the prognosis of HPV DNA+ HNCUP more closely resembled that observed in HPV DNA+ tonsillar and base‐of‐tongue OPSCC, with significantly improved survival outcomes compared with its HPV DNA‐ HNCUP counterpart, supporting the hypothesis that a substantial proportion of HNCUP represents occult OPSCC primaries [7]. In accordance with these and previous findings, HPV DNA+ HNCUP has been managed as HPV DNA+/p16+ OPSCC at Karolinska University Hospital since 2014, as described above.

Although the available literature on the prognostic significance of non‐HPV genotypes in OPSCC remains limited, the few published studies suggest that patients with HPV‐positive/non‐HPV16 OPSCC, including HPV33‐positive tumors, have more favorable clinical outcomes than those with HPV‐negative OPSCC [25, 26]. A similar pattern was observed in the present HNCUP cohort, where both HPV16+ and non‐HPV16+ tumors were associated with more favorable outcomes than HPV‐negative tumors. However, the number of non‐HPV16 cases was small, precluding more detailed genotype‐specific analyses or meaningful comparisons between individual HPV‐genotypes.

This study also has several limitations. Although, to our knowledge, it represents one of the largest reported HNCUP cohorts, the overall number of included patients remains limited due to the rarity of the disease, which accounts for less than 5% of all head and neck cancers [27, 28].

Furthermore, the retrospective design entails inherent limitations, including incomplete data capture, as a subset of HNCUP cases lacked p16 information, thereby reducing the number of cases eligible for combined biomarker analysis. In addition, changes in HPV DNA testing methods, p16 assessment, imaging techniques, staging, and treatment strategies occurred during the study period. To evaluate the potential impact of treatment era, we performed a sensitivity analysis comparing patients diagnosed during 2000–2013 and 2014–2024. The findings were consistent across both periods, supporting the robustness of the observed associations between HPV DNA/p16 status and outcomes. However, residual confounding related to temporal changes in management cannot be completely excluded.

Moreover, p16 expression was assessed on FNAC samples from patients diagnosed between 2014 and 2024 using antibodies primarily optimized for FFPE tissue, whereas FFPE samples were used for patients diagnosed between 2000 and 2013. These methodological differences may have influenced the accuracy of p16 assessment and may partly explain the higher proportion of HPV DNA+/p16‐ cases observed in the more recent cohort. Additionally, the small size of the discordant subgroups (HPV DNA+/p16‐ and HPV DNA−/p16+) limits the precision and interpretability of the corresponding hazard ratio estimates. Larger prospective studies are therefore warranted to validate and further refine these findings.

Another limitation is that detailed information regarding smoking exposure was not available. Although smoking status could be categorized as ever‐smoker or never‐smoker, data on cumulative exposure (pack‐years) and duration of smoking cessation were unavailable. Therefore, although ever‐smoking status was associated with DFS but not OS in the present study, further studies are needed to validate these findings and assess the potential impact of smoking intensity and duration of cessation.

Nevertheless, a notable strength of this study is that the Swedish healthcare system, based on universal health coverage, enables population‐based real‐world studies and ensures equitable access to care. Therefore, despite temporal changes in diagnostic and treatment strategies during the study period, patients were managed according to comparable standards of care, supporting the internal consistency of the cohort.

## 5 Conclusions

In this large HNCUP cohort from a population with high HPV prevalence and low‐smoking rate, combined assessment of HPV DNA and p16 provided superior prognostic stratification compared with either marker alone. HPV DNA positivity was consistently associated with favorable survival, and OS among HPV DNA+/p16‐ cases was comparable to that of HPV DNA+/p16+ cases when HPV‐DNA was considered as a single marker, although DFS estimates in this small subgroup were imprecise. In contrast, HPV DNA‐negative disease conferred poor prognosis irrespective of p16 status.

The extent of HPV DNA/p16 mismatch and the prognostic significance of p16 expression vary across HNCUP cohorts, underlining the need for biomarker strategies optimized for the specific clinical setting. These findings support the use of dual‐marker testing, whenever feasible, to ensure accurate biological classification and optimal clinical decision‐making. As HNCUP has become an increasingly rare entity due to advances in diagnostic techniques, future progress will likely depend on well‐designed multicentre studies to enable adequately powered analyses and further refine prognostic stratification.

## Author Contributions

**Evelina Jörtsö:** data curation, formal analysis, methodology, validation, visualization, writing – original draft, writing – review and editing. **Anders Näsman:** formal analysis, methodology, software, validation, writing – review and editing. **Igor Schliemann:** data curation, methodology, validation, writing – review and editing. **Mark Zupancic:** writing – review and editing. **Lars Sivars:** data curation, writing – review and editing. **Tina Dalianis:** formal analysis, writing – review and editing. **Linda Marklund:** conceptualization, methodology, project administration, funding acquisition, visualization, supervision, writing – review and editing, writing – original draft. **Rusana Bark:** conceptualization, data curation, methodology, formal analysis, project administration, funding acquisition, validation, visualization, supervision, writing – original draft, writing – review and editing.

## Funding

This work was supported by ACTA Otolaryngologica Foundation.

## Ethics Statement

This study was approved by The Swedish Ethical Review Authority (Ethical numbers: 2009/1278‐31/4, 2018/870‐32, 2023‐04595‐01 and 2023‐05249‐01). Data were retrieved from medical records in compliance with ethical permissions above. In accordance with the approval of the Regional Ethics Committee, the requirement for informed consent was waived, as the study involved only retrospective analyses.

## Conflicts of Interest

The authors declare no conflicts of interest.

## Data Availability

The dataset used in this study contains pseudonymized (coded) personal data derived from medical records and cannot be made publicly available. Public sharing would conflict with the study's ethical approval and may increase the risk of participant re‐identification due to the small and clinically specific study population. The key linking the codes to identifiable individuals is retained by the health care provider. In accordance with the EU General Data Protection Regulation (GDPR) and the approval of the Swedish Ethical Review Authority, access to the data is restricted. Data may be made available for non‐commercial research purposes upon reasonable request and under a data access agreement ensuring compliance with data protection, ethical, and institutional requirements. Requests should be directed to the Research Data Office at Karolinska Institutet (rdo@ki.se), which will manage the legal and ethical review and coordinate secure data transfer if approved.
